# Diagnosis and Treatment of Iron Deficiency and Iron Deficiency Anemia in Children and Adolescents: Recommendations of the Polish Pediatric Society, the Polish Society of Pediatric Oncology and Hematology, the Polish Society of Neonatology, and the Polish Society of Family Medicine

**DOI:** 10.3390/nu16213623

**Published:** 2024-10-24

**Authors:** Radosław Chaber, Ewa Helwich, Ryszard Lauterbach, Agnieszka Mastalerz-Migas, Michał Matysiak, Jarosław Peregud-Pogorzelski, Jan Styczyński, Tomasz Szczepański, Teresa Jackowska

**Affiliations:** 1 Department of Pediatrics, Institute of Medical Sciences, University of Rzeszow, 35-310 Rzeszow, Poland; 2 Clinic of Pediatric Oncology and Hematology, State Hospital 2, 35-301 Rzeszow, Poland; 3 Department of Neonatology and Neonatal Intensive Care, Institute of Mother and Child, 04-370 Warsaw, Poland; e.helwich@gmail.com; 4 Clinic of Neonatology, Department of Gynecology and Obstetrics, Jagiellonian University Hospital, 31-501 Cracow, Poland; ryszard@lauterbach.pl; 5 Department of Family Medicine, Wroclaw Medical University, 51-141 Wroclaw, Poland; agnieszka.migas@gmail.com; 6 Department of Oncology, Children’s Hematology, Clinical Transplantology and Pediatrics, University Clinical Center, Medical University of Warsaw, 02-091 Warsaw, Poland; mmatysiak@interia.eu; 7 Department of Pediatrics, Oncology and Pediatric Immunology, Pomeranian Medical University, 70-204 Szczecin, Poland; jwperegud@gmail.com; 8 Department of Pediatric Haematology and Oncology, Collegium Medicum, Nicolaus Copernicus University Torun, Jurasz University Hospital 1, 85-094 Bydgoszcz, Poland; jstyczynski@cm.umk.pl; 9 Department of Pediatric Haematology and Oncology, Medical University of Silesia, 41-800 Katowice, Poland; szczep57@poczta.onet.pl; 10 Department of Pediatrics, Centre of Postgraduate Medical Education, 01-809 Warsaw, Poland; tjackowska@gmail.com

**Keywords:** iron deficiency, anemia, children, diagnosis, prevention, treatment

## Abstract

**Background/Objectives.** Iron deficiency is one of the most common nutritional deficiencies worldwide and is the leading cause of anemia in the pediatric population (microcytic, hypochromic anemia due to iron deficiency). Moreover, untreated iron deficiency can lead to various systemic consequences and can disrupt the child’s development. **Methods/Results.** Therefore, a team of experts from the Polish Pediatric Society, the Polish Society of Pediatric Oncology and Hematology, the Polish Neonatology Society, and the Polish Society of Family Medicine, based on a review of the current literature, their own clinical experience, and critical discussion, has developed updated guidelines for the diagnosis, prevention, and treatment of iron deficiency in children from birth to 18 years of age. These recommendations apply to the general population and do not take into account the specifics of individual conditions and diseases.

## 1. Introduction

Individual nutrient deficiencies are an important factor in the overall health of children and the incidence of many diseases. The youngest children in particular, during the period of intensive growth and development, are vulnerable to the effects of deficiencies, which can be irreversible [1]. Iron deficiency (ID) is one of the most common nutritional deficiencies worldwide, which—according to the World Health Organization (WHO)—affects approximately 2 billion people [2,3], mainly pregnant women, fetuses, and children. It is also the most common cause of anemia [3,4,5,6,7,8]. The prevalence of ID in Polish children is unknown. In Europe, it is estimated at <2% of infants up to 6 months of age [9], 4–18% of infants aged 6–12 months [10], 4–41% of children aged 1–3 years [10], approx. 2–6% of pre-school children [9], and approx. 8–20% of adolescent girls [11]. Its incidence is significantly affected by socioeconomic conditions [12]. The most important complication of ID, iron deficiency anemia (IDA), is significantly more common in Eastern European countries (9–50%) than in Western and Northern Europe (<5%) [10].

Iron, due to its unique chemical properties (ease of entering oxidation–reduction reactions: Fe^2+^/Fe^3+^), plays a key role in many biological processes: energy production, oxygen transport, immune reactions, biomolecule synthesis, etc. For this reason, chronic deficiency of this element, especially during periods of intense growth and development, can have serious systemic consequences. Microcytic anemia is an important and relatively late-occurring result of ID, starting from intrauterine development onwards, albeit it is not the only one [13]. Impaired psychomotor and intellectual development can occur even in children with chronic iron deficiency without accompanying anemia and can be permanent [14,15,16,17,18,19,20]; however, there is still no conclusive evidence to support this [21], and the results of studies to date are also inconclusive [22].

This does not change the fact that the identification of populations of children at risk of iron deficiency and early iron supplementation in these groups is crucial from a public health perspective. Taking into account the great importance and scale of the problem of iron deficiency in the pediatric population, the Polish Pediatric Society, the Polish Society of Pediatric Oncology and Hematology, the Polish Society of Neonatology, and the Polish Society of Family Medicine set up a joint expert team to develop current guidelines for the diagnosis, prevention, and treatment of iron deficiency in children from birth to 18 years of age based on a review of the current literature, their own clinical experience, and critical discussion. The following recommendations apply to the general population and do not take into account the specifics of individual diseases and conditions.

## 2. Causes of Iron Deficiency

In the pediatric population, ID/IDA are most common in three age groups:

(1) Infants, in particular premature babies, especially after 6 months of age;
(2) Pre-school children;
(3) Teenage girls.

They develop through two basic mechanisms:

(1) Insufficient supply and/or absorption of iron from the gastrointestinal tract;
(2) Blood loss that exceeds the ability to replenish dietary iron loss.

The most important causes of ID in children are summarized in Table 1 [23,24,25,26,27].

All children diagnosed with the conditions listed in Table 1 and/or who follow a low-iron diet (e.g., vegetarian, vegan, cow’s milk-rich diet) are at risk of iron deficiency. This risk increases particularly in states of increased demand for this element [25]:

- During the period of rapid growth in infants, especially those born prematurely;
- During the period of rapid growth at puberty;
- In children involved in competitive sports.

In infancy, ID may be due to lower iron stores accumulated during fetal life, which are used up until iron-rich foods, especially meat, are included in the diet. Moreover, the following groups are particularly at risk of iron deficiency in infancy [25,28]:

- Children born prematurely, especially those who were treated with erythropoietin without a concomitant sufficient iron supply [29];
- Children from multiple pregnancies;
- Newborns who are too small for their gestational age (2000–2500 g);
- Children of mothers who had anemia during pregnancy [30];
- Infants fed with unmodified cow’s milk before 12 months of age [31,32,33,34];
- Children with perinatal blood loss, including the following:
  – hemorrhaging or bleeding.
  – frequent blood sampling in a newborn.
  – fetal–maternal leakage (placenta previa, placental abruption, cesarean section, amniocentesis, cordocentesis, abdominal trauma of the pregnant woman).
  – twin-to-twin transfusion syndrome.

Dietary iron absorption occurs mainly in the duodenum [27]. Impaired iron absorption from the gastrointestinal tract occurs in diseases that affect this part of the intestine: celiac disease [35], Crohn’s disease [36], giardiasis [37], and resection of the proximal small intestine [38]. In children, anemia secondary to malabsorption of iron, folic acid, and vitamin B12 is a common complication of celiac disease [39] and inflammatory bowel disease [40,41].

In addition to malabsorption, ID is exacerbated by chronic gastrointestinal (GI) bleeding associated with various GI pathologies. These include, but are not limited to, enteropathy, which often occurs in young children fed unmodified cow’s milk [42], food allergy [43], and inflammatory bowel disease—Crohn’s disease and ulcerative colitis [44,45].

Chronic blood loss is the predominant cause of ID in adolescent girls with prolonged and/or heavy menstrual bleeding. It is estimated that this problem affects approximately 18–38% of women of reproductive age [46], and ID occurs in approximately 30–50% of girls with this symptom [47,48].

Another group of ID causes involves abnormal secretion of hepcidin, which is a hormone produced by the liver, also as an acute phase protein. Hepcidin is responsible for the regulation of systemic iron homeostasis. By binding to ferroportin, an iron-transporting protein, e.g., from enterocytes and macrophages of the reticuloendothelial system of the spleen, it inhibits the supply of iron from the GI tract and iron from erythrocytes destroyed in the spleen [49,50]. Chronic inflammation, accompanying, e.g., infection, autoimmune disease, or allergy, increases hepcidin secretion in the liver, resulting in ID [51]. Increased hepcidin secretion has also been shown to occur in obese children [52,53,54], which, together with increased iron requirements, may lead to the deficiency of this micronutrient in this pediatric population [55,56,57]. Obesity nearly doubles the risk of developing ID [(odds ratio—OR): 2.1; 95% confidence interval (CI): 1.4–3.2] [58]. In addition, ID is approximately three times more common in obese girls than in obese boys [55,56].

The specific risk groups for iron deficiency are listed in Table 2.

## 3. Symptoms and Consequences of Iron Deficiency

Due to the essential role of iron in metabolism, iron deficiency affects the functioning of the entire body, especially organs and tissues with high energy demand, e.g., the central nervous system, hematopoietic system, and epithelial tissue. The developing anemia and associated hypoxia further disrupt cellular homeostasis, which can exacerbate already existing symptoms of ID and cause anemia-specific symptoms, regardless of its cause.

The ID and IDA symptomatology is dependent on the following [59]:

- Severity of ID and hemoglobin concentration;
- Timing of ID and/or IDA onset and the potential to generate adaptive mechanisms;
- Age of the child;
- Current iron needs;
- Presence of comorbidities.

The symptoms of ID and IDA are summarized in Table 3.

## 4. Laboratory Diagnosis of Iron Deficiency

Approximately 75% of the body’s total iron content is bound in the oxygen-transporting hemoproteins, hemoglobin and myoglobin. The rest is contained in iron storage proteins, mainly in the liver, i.e., ferritin and hemosiderin, which also bind excess iron in the cell, protecting it from its toxic effects. A small proportion of iron (approx. 3%) is used by various enzymes (including mitochondrial enzymes responsible for energy production) as a cofactor [15,95].

### 4.1. Serum Ferritin Levels

Reduced serum ferritin (SF) is now considered to be the best and most specific marker of iron availability in the body, and its determination can be used in routine diagnosis. Ferritin levels are proportional to the total amount of iron stores [96,97], so this parameter can be used to diagnose both iron deficiency and also iron excess in the body [98,99,100,101]. In addition, the low cost of measuring SF levels and the small volume of blood required for this assay support the widespread use of SF. The disadvantage of this assay is its dependence on the presence of concurrent inflammation (ferritin is an acute-phase protein); therefore, higher SF values should be adopted as cut-off points for the diagnosis of ID in the event of concurrent inflammation or active infection. Higher SF values may persist even for several weeks after the inflammation has passed [102]. Following strenuous exercise, elevated SF levels may persist for several days [103]. Ferritin can be measured in commercial laboratories using a number of automated methods, including immunoturbidimetry and latex agglutination. At present, discrepancies in the obtained SF values depending on the assay method, postulated by some authors, do not seem to be of significant importance [104,105]. Instrument calibration using manufacturer-supplied standards, which are themselves calibrated against international reference standards, and quality assurance activities ensure that the results obtained are within a similar range to those reported by other laboratories [98]. The 2018 systematic review by Garcia-Casal et al. [106] concluded that the laboratory methods most frequently used to determine ferritin concentrations have comparable accuracy and performance.

It is also important to note that, in the pediatric population, physiological SF values are characterized by high variability depending on the child’s age, especially in the first two years of life [107]. To date, there have been no universally accepted standardized SF normal ranges. According to the current recommendations of 2020, the WHO defines ID as SF < 12 µg/L in children under 5 years of age and <15 µg/L in children over 5 years of age. Identical values are also the cut-off point for the diagnosis of ID according to the 2021 British Society for Haematology (BSH) recommendations [108]. In the case of concurrent inflammation [present signs and symptoms of inflammation or elevated inflammatory markers, e.g., C-reactive protein (CRP)], these values increase to SF < 30 µg/L in children under 5 years of age and <70 µg/L in children over 5 years of age [109]. The results of some studies justify raising the lower limit of SF levels to 18–24 µg/L as this is deemed more adequate for determining sufficient iron stores in the body [110,111,112]. In contrast, according to the 2020 recommendations of the Swiss Pediatric Oncology Group (SPOG) [66], SF values as low as <10 µg/L justify the diagnosis of ID in most age groups, whereas the 2019 standards published by the American Academy of Pediatrics (AAP) [113] define this value as <7 µg/L (although the AAP recommendations for children aged 0–3 years [14] set this level at 10–12 µg/L).

Due to large discrepancies regarding the lower limit of normal SF, especially for children up to 24 months of age, further studies—also of the Polish population—are needed to determine the lower limit of SF levels in individual age groups, ensuring normal body development and function in the developmental period.

For use in daily clinical practice, we currently recommend standards in line with the Swiss Pediatric Oncology Group (SPOG) guidelines [66], which are summarized for each age range in Table 4.

Given the significant impact of infection/inflammation on SF levels, simultaneous determination of CRP levels or another inflammatory marker, e.g., erythrocyte sedimentation rate (ESR) and fibrinogen, is indicated in cases where infection/inflammation cannot be definitely excluded on the basis of anamnesis and physical examination.

### 4.2. Peripheral Blood Count

The inevitable consequence of an uncontrolled ID is IDA. For this reason, peripheral blood count should be performed as part of the standard diagnosis of ID. Erythrocytes in IDA are characterized by lower mean corpuscular volume (MCV), lower mean corpuscular hemoglobin (MCH), and lower mean corpuscular hemoglobin concentration (MCHC). The red blood cell distribution width (RDW) determines the degree of variation in erythrocyte size (degree of anisocytosis). The higher the RDW value, the greater the variability in erythrocyte size. RDW is the first morphological parameter that changes in anisocytosis, which is associated, among other things, with ID. The correct RDW value expressed in percentage as the red blood cell distribution width–coefficient of variation (RDW-CV) is 11.5–14.5% [114]; it can also be expressed as the red blood cell distribution width–standard deviation (RDW-SD). In ID/IDA, the RDW value is elevated, in contrast to another microcytic anemia—thalassemia, characterized by a homogeneous microerythrocyte population (RDW is then normal). When thalassemia is excluded, low MCV, MCH, and MCHC values are strongly suggestive of IDA, while normal values for these parameters do not exclude ID as a cause of anemia. In the latter case, further investigations are needed to confirm or exclude ID [108].

The physiological values of hemoglobin (Hb) concentration and the above blood cell parameters differ significantly between different age groups of children. According to the World Health Organization (WHO) [115], the average Hb value in children aged 6–59 months in Poland should be 12.0 g/dL (range: 11.3–12.5), but there are no universal standards for other age groups. For this reason, we recommend the normal ranges for hemoglobin concentration and MCV according to the Swiss Pediatric Oncology Group (SPOG) guidelines [66]—Table 4.

When anemia of unclear etiology is suspected, the complete blood count should be expanded to include the white blood cell differential and reticulocyte count. In IDA, the reticulocyte count may initially remain normal; however, it decreases significantly over time. Reticulocyte count measurement by modern analyzers also provides information on the hemoglobin content of reticulocytes (abbreviated ‘RETHe’ in Sysmex analyzers, ‘CHr’ in Siemens Advia, ‘MCHr’ in Abbott Sapphire); it is an early marker of erythropoietic activity. This parameter can be regarded as a real-time functional marker of ID (reticulocytes remain in the blood for only 1–2 days) [116,117]. Important advantages of this test are its high sensitivity, no reaction to inflammation, and relatively low cost of performance [14,118,119]. The CHr value < 27.5 pg has high specificity and sensitivity for the diagnosis of iron deficiency in infants and young children, as does CHr < 28 pg in older children and adults [120,121,122,123,124]. The above values do not apply to patients with thalassemia, in which the CHr parameter is decreased. According to a 2022 meta-analysis [125], the reticulocyte count may be more valuable in the diagnosis of IDA than MCV and SF. Furthermore, CHr together with other red blood cell parameters can be used to differentiate beta-thalassemia from IDA [126,127].

### 4.3. Other Assays

Free serum iron (Fe), total iron-binding capacity (TIBC), and unsaturated iron-binding capacity (UIBC), as well as transferrin saturation (TfS), should not be decisive at present, but only complementary. These parameters, like SF, are poor indicators of iron deficiency in inflammatory or infectious conditions and are also characterized by high diurnal variability [128] and depend on the type of food. These assays can be used to control compliance, i.e., adherence to the doctor’s instructions, or when an overdose of an iron preparation is suspected [129]. TfS values <16% [TfS (%) = (iron × 100)/TIBC] may suggest ID if other assays are inconclusive [108].

Soluble transferrin receptor (sTfR) is an extracellular fragment of the transferrin receptor whose serum concentration increases in a state of functional iron deficiency [130]. Its advantage, i.e., the fact that serum sTfR levels are not much affected by inflammation [59], does not outweigh its disadvantages such as lack of standardization assays [131], high cost, insufficient sensitivity in the early phases of ID [132], and increased sTfR levels in states of stimulated erythropoiesis such as hemolysis, megaloblastic anemia, thalassemia, and hypoxia [133]. For the above reasons and due to the limited availability of this test, determination of sTfR for the diagnosis of ID is not widely recommended [108], unless there is a significant need to diagnose ID associated with infection/inflammation [6,109].

In iron deficiency, a zinc atom is incorporated into protoporphyrin IX instead of iron, resulting in an increased concentration of zinc protoporphyrin (ZPP) in erythrocytes. This test has a number of limitations, e.g., false high values are detected with concomitant inflammation, hemoglobinopathy [98], hyperbilirubinemia [134], and chronic kidney disease [135]. Its advantage is the small volume of blood sample needed—0.2 mL. Low specificity and low availability significantly limit the use of this assay in routine diagnosis [108], although its use in uncertain and inconclusive cases may be considered [14,136].

Hepcidin—the test is not yet standardized—has a high cost and is not available for routine diagnosis [137]. For this reason, its performance is not recommended [108].

## 5. Monitoring Iron Deficiency/Iron Deficiency Anemia

Modern analytical methods make it possible to detect iron deficiency in the body at quite an early stage, even in the latent phase of deficiency, i.e., before significant clinical symptoms occur. Screening for risk factors and symptoms of ID/IDA should be carried out in children at every possible visit, especially up to the age of 3 years. According to one approved schedule, such assessments should be conducted at 4, 15, 18, 24, 30, and 36 months of age, and once a year thereafter. Early identification of ID risk factors and symptoms is more important for the prevention of IDA than routine laboratory testing [138].

Laboratory diagnostics to assess iron status (primarily SF) and complete blood counts are indicated if typical symptoms of ID/IDA are observed during anamnesis and physical examination. In these cases, testing should not be delayed in order to start supplementation as early as possible and prevent serious deficiency of this element.

Currently, there is no conclusive evidence to support the benefit of routine monitoring of complete blood count and iron status in children without symptoms of ID/IDA [21]. However, some guidelines recommend prophylactic measurement of complete blood count (CBC) in every child. According to the American Academy of Pediatrics (AAP) [14,139], a complete blood count should be performed around 12 months of age, and according to the Centers for Disease Control and Prevention (CDC) [140], at 9–12 months of age, then at 15–18 months of age, and annually thereafter, until the age of 5 (in populations at high risk of ID). However, according to most other guidelines [e.g., U.S. Preventive Services Task Force (USPSTF) [141], Spanish Association of Primary Care Paediatrics (PrevInfad/PAPPS) [142], United Kingdom National Screening Committee (UK NSC) [143], European Society for Pediatric Gastroenterology, Hepatology, and Nutrition (ESPGHAN) [9], and the Swiss Pediatric Oncology Group (SPOG) [66]], such a procedure is not justified, although it is not contraindicated either. Periodic laboratory tests should be considered especially in children who have been treated with iron preparations in the past and who still have risk factors for ID (e.g., poor diet, heavy and prolonged menstrual bleeding). ID risk groups among children are summarized in Table 2. Regular monitoring of iron levels in these children will help avoid significant fluctuations in bioavailable iron levels in the body in a situation where clinical symptoms occur relatively late, already with a significant deficiency of this element. In these groups, routine laboratory tests (CBC, SF) should be considered at 15–18 months of age [107], during early childhood (2–5 years of age), and thereafter depending on individual indications. In adolescents, routine laboratory screening should be conducted for girls after menarche and at least every 5 years thereafter, and for boys, at least once during the period of rapid growth in the pubertal spurt. In adolescents with risk factors (especially those with a history of ID/IDA, those on a low-iron diet, and girls with heavy/prolonged menstrual bleeding), laboratory tests should be performed more frequently [144].

## 6. Treatment of Iron Deficiency/Iron Deficiency Anemia

Effective treatment of ID/IDA should be based on the following:

- Dietary recommendations;
- Oral, or possibly intravenous, supplementation of this element;
- Monitoring response to treatment.

### 6.1. Dietary Recommendations

Infants up to 6 months of age with ID/IDA who are not breastfed should receive iron-fortified formula, with an iron content of approx. 4–8 mg/L. Infants over 6 months of age who are not breastfed should receive iron-fortified follow-on formulas; however, the optimal iron content of these products has not been clearly established [9]. Solid iron-rich foods should be used for the nutrition of all children over 6 months of age. Foods with the highest iron content are meats (bioavailability of approx. 20–25%) such as liver (pork or veal), beef or pork, game, as well as egg yolk, trout, and other fish; plant foods with the highest iron content (bioavailability of approx. 5–10%) include wheat bran, sesame seeds, soya beans, lentils, millet groats, white beans, dried apricots, spinach (value limited due to the presence of oxalates), and whole grain bread [9]. In addition, foods that lower gastric pH and, thus, facilitate the absorption of iron, can be recommended. These include foods high in vitamin C, including orange juice, lemon juice, grapefruit juice, or other fruits—apples, grapes, gooseberries, pears, or raspberries [66]. Foods with the highest iron content are listed in Table 5. Milk intake should also be limited to a maximum of 500 mL/day [9,145,146,147]. Children up to 12 months of age should not be given unmodified cow’s or goat’s milk, which can contribute to the development of enteropathy and associated malabsorption as well as bleeding leading to iron loss [9,66,148]. Furthermore, it is recommended to limit the intake of products containing iron absorption inhibitors: polyphenols (tea [149]), phytates, oxalates, etc., which are present in large quantities in vegetarian diets.

### 6.2. Oral Iron Supplementation

The best and the most physiological method of iron supplementation is oral intake. For iron supplementation, there are many different chemical formulations of iron (simple iron salts and complex compounds with modified and extended release) available now. To date, there are no reliable studies in the pediatric population evaluating different chemical formulations of iron preparations, their dosages, and the duration of therapy [150].

The ideal dose of iron should be calculated in terms of elemental iron. The total daily therapeutic dose of elemental iron is 3–6 mg/kg of body weight (BW) in 1–3 divided doses. Considering the physiology of iron absorption and the results of recent studies [151,152,153], administration of high doses of iron in 2–3 doses per day is deemed ineffective. The best iron absorption is achieved with intermediate doses and when administered every other day [151]. This dosage is recommended for patients with mild symptoms of ID, no IDA, or mild IDA. However, high doses of iron increase absolute iron absorption; therefore, higher doses may be considered in cases of severe ID or iron malabsorption [24]. The maximum daily dose is 150–200 mg of elemental iron [154]. An iron dose of 3 mg/kg BW is as effective as higher doses [151,155,156,157,158,159], and it rarely causes gastrointestinal adverse effects [160,161,162,163,164,165]. A single daily dose given before bedtime is as well tolerated as dosing in three divided doses [166], and it may contribute to better compliance and may result in fewer adverse effects compared to administering larger doses and splitting the daily dose into a greater number of divided doses. Hepcidin levels increase after oral iron administration for up to 24 h, so iron absorption is greatest with a single daily dose (compared to two or more doses) [155]. Moreover, iron administration on an empty stomach in the evening can minimize gastrointestinal discomfort, while reduced gastrointestinal motility during sleep increases iron absorption [167].

In addition to elemental (carbonyl) iron [168,169], bivalent and trivalent iron salts are commonly used; they demonstrate similar activity, but should be administered in different doses [66,170,171]:

- Fe^2+^ salts: 2–3 mg/kg BW in one or two doses/day, half an hour before a meal or half an hour after a meal; juice or water can be used to improve the taste;
- Fe^3+^ salts: 3–5 mg/kg BW in one or two doses/day, with meals (preferably with juice or water; polymaltose is a sugar complex and must be dissolved in gastric juice in order for the iron to be available in the intestine).

Other preparations include iron chelates, iron hydroxides, iron–polysaccharide complexes, and heme iron. The iron preparations for children, available in Poland, are summarized in Table 6.

In children, adverse effects after oral iron intake at the doses given above are not common [15,155]. They mostly include gastrointestinal complications such as abdominal pain, nausea, vomiting, constipation or diarrhea (severity depends on the type of preparation), feeling of fullness, darkened teeth (after administration of some preparations of Fe^2+^ salts, it occurs temporarily during treatment; use of neutral liquids or rinsing the mouth after administration of the drug prevents discoloration), and a metallic taste in the mouth. In addition, black stool is observed due to the presence of iron sulfides, whereas the absence of the black color indicates irregular use of the drug [165]. In the event of adverse reactions that prevent iron intake at the recommended dose, the following may be considered:

- Change of product, especially Fe^2+^ salts to Fe^3+^ [66];
- Change of the iron preparation formula, e.g., drops to suspension and vice versa;
- Administration of an ideal iron dose every other day, which helps to avoid peak hepcidin secretion [151,152,153,172] so that iron absorption is greatest and equivalent to daily supplementation. There is currently no evidence of the efficacy of such treatment in the pediatric population;
- Switching to parenteral supplementation.

### 6.3. Assessment of Responses to Oral Iron Treatment

The first sign of the efficacy of iron supplementation is an increase in the reticulocyte count, observed from day 3 to day 4 of treatment and persisting for about 2 weeks. Hemoglobin concentration starts to increase after about two weeks of therapy [173]. An analysis of five randomized trials showed that the absence of an increase in hemoglobin > 1.0 g/dL on day 14 of oral iron administration usually indicates the need for intravenous treatment [174].

Iron is usually administered orally for approximately 3 months after the Hb level has normalized [66,129,173,175]. The above duration of iron administration has not been conclusively established by scientific evidence [150,155], and in some cases, replenishment of iron stores and full normalization of serum SF may require up to 6 months of treatment [176].

The achievement of the above criteria for Hb increase confirms a diagnosis of IDA, whereas a lack of Hb increase after 2–4 weeks of iron supplementation may be due to the following reasons [175]:

(1) Non-compliance—no or irregular intake of iron; absence of stool darkening may indicate failure to take iron;
(2) Inadequate iron dose;
(3) Ineffective iron preparation;
(4) Insufficient duration of therapy;
(5) Persistent or undiagnosed blood loss, e.g., Meckel’s diverticulum;
(6) Incorrect diagnosis (e.g., thalassemia, sideroblastic anemia);
(7) Impaired iron absorption or utilization (e.g., chronic inflammation, infection, celiac disease, inflammatory bowel disease, cancer, liver or kidney disease, concomitant vitamin B12 and folic acid deficiencies, thyroid disease, lead poisoning);
(8) Impaired absorption from the gastrointestinal tract due to high gastric pH (e.g., antacids, histamine 2 blockers, proton pump inhibitors), Helicobacter pylori infection [177] (competes for available iron);
(9) Hereditary iron refractory iron deficiency anemia (IRIDA);
(10) Other rare genetic disorders of iron transport.

In 2–5% of patients with IDA of unclear origin, celiac disease is diagnosed [178]; therefore, this disease must be excluded in any child with refractory and recurrent IDA, especially of unclear etiology [179,180,181]. The preferred screening test for celiac disease is the tissue transglutaminase IgA (tTG-IgA) assay, which has the highest sensitivity, together with the measurement of IgA levels, or an IgG-based assay (2–3% of patients with celiac disease are IgA-deficient). Following positive serological tests, celiac disease should be confirmed by small intestine assessment [179].

### 6.4. Parenteral Iron Supplementation

At present, severe symptomatic IDA, even with low hemoglobin levels, is not an absolute indication for parenteral supplementation [182]. A rapid and good response to oral iron administration also occurs at low hemoglobin concentrations [158,159]. In contrast, a meta-analysis of 72 studies involving 10,605 patients [183] found that intravenous iron therapy was more effective in increasing hemoglobin levels and reducing the risk of transfusion than its oral supply; however, this possible benefit was offset by an increased risk of infection.

In children, intravenous iron infusion should now be the only form of parenteral treatment. Intramuscular injections are associated with significant pain, skin discoloration, and the risk of soft tissue necrosis at the injection site [66].

Indications for parenteral iron administration are limited to the following [25,66,175,184]:

- Lack of response to high-dose oral iron treatment with at least two different preparations;
- Oral treatment intolerance;
- Non-compliance with orders;
- Severe chronic bleeding, with blood loss too high to be compensated by oral iron intake, e.g., heavy and prolonged menstrual bleeding;
- IRIDA;
- Need for rapid iron replenishment, e.g., erythropoietin treatment, circulatory failure;
- GI diseases that prevent iron absorption;
- Chronic kidney disease.

The advantage of parenteral iron substitution is that gastrointestinal adverse effects are avoided and the iron dose can be accurately calculated to achieve the desired hemoglobin concentration. Its drawbacks are significant limitations, a greater impact on the child (venipuncture, hospitalization), and significantly higher costs [9].

For parenteral supply, the following are currently available in Poland: iron (III) hydroxide–sucrose complex (approved for children), iron (III) hydroxide–dextran complex (approved for children over 14 years of age), iron (III) derisomaltose (not approved for children under 18 years of age). The dose of iron needed for intravenous (IV) administration is calculated using the Ganzoni formula [185,186]:

Total Fe deficiency [mg] (total cumulative dose) = body weight [kg] × (target Hb value − actual Hb value) [g/dL] × 2.4 + depot iron need (amount of Fe needed to replenish the reserves),

where target Hb and depot iron needs are as follows:

- Hb 13 g/dL, depot iron needs 15 mg/kg BW for children weighing <35 kg,
- Hb 15 g/dL, depot iron needs 500 mg BW for children weighing >35 kg.

The first generation of parenteral iron products [based on high-molecular-weight iron dextran (HMWID)] was associated with serious anaphylactic reactions and should no longer be used in children [187]. Newer products, including iron (III) derisomaltose and iron (III) hydroxide–sucrose complex, bind iron more tightly and release it more slowly, thus the risk of serious or severe anaphylactic reactions is currently not high [188,189,190,191]. Early adverse effects of intravenous iron infusion include nausea, vomiting, headache, erythema, myalgia, itching, joint pain, back pain, and chest pain. Laboratory tests sometimes show transient hypophosphatemia [188]. Skin discoloration after extravasation may also occur [192].

Due to the improved safety of the new intravenous iron preparations, they should be used more frequently in children in cases where substitution with an oral preparation is impossible or ineffective [189,193].

### 6.5. Transfusion of Red Cell Concentrate

The decision to perform red cell concentrate transfusion should be based primarily on the overall clinical context—the child’s general condition, comorbidities, adaptation to anemia, laboratory test results, etc.—and the risks, benefits, and possible alternatives to transfusion. Even low hemoglobin levels do not justify transfusion as long as the patient is in good general condition and does not show symptoms of circulatory insufficiency [138,175]. According to current recommendations [194], RBC (red blood cell) transfusion in critically ill children or those at risk of critical illness is indicated if Hb is <5 g/dL. Therefore, this procedure is rarely justified in most patients, even those with severe IDA. The red cell concentrate transfusion standard recommends a slow transfusion of 5 mL/kg BW/hour over 4 h (unless there is severe bleeding) to avoid complications [195].

## 7. Prevention of Iron Deficiency in Children

1. Given that 60–80% of total iron stores in newborns come from the third trimester of pregnancy [14,196], it seems reasonable to compensate for maternal ID during pregnancy. This is consistent with the WHO recommendation for prophylactic iron substitution in pregnant women living in areas with a high prevalence of ID [197]. However, according to the U.S. Preventive Services Task Force (USPSTF) and ESPGHAN CoN, there is insufficient evidence to recommend screening or treatment of iron deficiency anemia in pregnant women to improve iron status parameters in newborns [198]. Two Cochrane reviews [199,200] have shown that iron supplementation before delivery increases maternal hemoglobin concentration, but this is not associated with statistically significant clinical benefits, e.g., low birth weight, preterm birth, infections, and postpartum hemorrhage, for the newborn.
2. Delayed umbilical cord clamping (>120–180 s) after birth reduces the risk of ID/IDA in infants, especially those born prematurely and small for their gestational age [191,201,202,203,204].
3. Iron supplementation in low-birth-weight infants should be as follows [175]:
  - Infants weighing 1.5–2.0 kg: 2 mg/kg BW/day of elemental iron;
  - Infants weighing 1.0–1.5 kg: 3 mg/kg BW/day of elemental iron;
  - Infants < 1 kg: 4 mg/kg BW/day of elemental iron;
  - In turn, according to the ESPHAGAN CoN recommendations [9]:
  - Newborns and infants with low birth weight (2000–2500 g) should receive iron preparations at a dose of 1–2 mg/kg BW/day, starting at 2–6 weeks of age, until 6 months of age, regardless of whether they are pre-term or full-term newborns/infants,
  - Newborns and infants with a birth weight of <2000 g should receive iron preparations at a dose of 2–3 mg/kg BW according to the ESPGHAN guidelines for enteral nutrition in preterm infants.

Conclusions of the 2019 systematic review of intervention studies (McCarthy et al.) [205] indicate that long-term iron supplementation increases hemoglobin and ferritin concentrations and decreases the incidence of ID/IDA in preterm and low-birth-weight infants. However, there is no high-quality evidence for the long-term effects of iron supplementation on the physical and neurological development parameters in this pediatric population. The problem of potential iron overload was also the most frequently ignored issue in the studies analyzed. Well-designed, long-term, randomized controlled trials (RCTs) are essential to determine the optimal iron dose and route of administration in neonates and infants born prematurely, taking into account short- and long-term health effects.

Results from another meta-analysis and a 2022 systematic review (Manapurath et al.) [206] showed that enteral iron supplementation had a small effect on the risk of infections and necrotizing enterocolitis, but the quality of evidence was low. An increase in body length was observed (moderate-quality evidence, mean difference 0.69 cm, 95% CI 0.01–1.37, I2 = 0%), but with little effect on weight, head circumference, or cognitive development. Two studies demonstrated an improvement in the risk of anemia (moderately confident evidence, RR 0.25, 95% CI 0.10–0.62, I2 = 0,00%), but no effect on serum ferritin levels was observed. Limitations of this meta-analysis included the heterogeneity of the reviewed studies.

4. According to the 2010 AAP recommendations [14], infants born at term and fed exclusively or at least at 50% with breast milk should receive iron supplementation at a dose of 1 mg/kg BW/day (maximum 15 mg) starting at 4 months of age until appropriate iron-containing complementary foods are introduced. In formula-fed infants, the iron contained in the formula (on average 10–12 mg/L) should meet their needs. Preterm babies should receive iron from 2 weeks of age (2–4 mg/kg BW/day, maximum 15 mg) until 12 months of age in the form of fortified formulas or an iron preparation. In contrast, ESPGHAN CoN [199] does not recommend iron supplementation in the general population of European breastfed infants over the age of 4–6 months. However, prophylactic iron supplementation may be recommended for infants at high risk of ID/IDA if the infant demonstrates a low intake of iron-rich complementary foods.
5. In populations with relatively low incidences of iron deficiency, there is limited evidence to show that routine iron supplementation is beneficial in healthy children aged > 6 months [207].
6. In accordance with the current strategy for the prevention of ID/IDA adopted in Poland [28], prophylactic iron is administered at a dose of 1–2 mg Fe/kg BW/day from 3 months of age until 1 year of age in the following groups:
  - Absolute indications:
    – preterm babies;
    – full-term babies with low birth weight (2000–2500 g);
    – children from multiple pregnancies;
    – children with reduced hemoglobin levels in the neonatal period;
    – children at risk of perinatal blood loss;
    – children of mothers with anemia during pregnancy.
  - Relative indications:
    – recurrent respiratory and gastrointestinal infections;
    – period of rapid growth;
    – impaired appetite;
    – bleeding tendency/heavy menstruation in pubertal girls;
    – overweight or obese children.
7. The dietary recommendations [157] related to the prevention of ID/IDA in infants and children are as follows:
  - Recommended dietary allowance (RDA) for iron [14,140,157,208]:
    – newborns and infants born prematurely: 2–4 mg/kg BW/day (maximum 15 mg);
    – newborns and infants carried to term: 1 mg/kg BW/day (maximum 15 mg);
    – 1–3 years of age: 7 mg/day;
    – 4–8 years of age: 10 mg/day;
    – 9–13 years of age: 8 mg/day;
    – adolescents aged 14–18 years: 11 mg per day for boys and 15 mg per day for girls;
    – pregnant girls: 27 mg/day.
  - Exclusive breastfeeding for the first six months of life [197];
  - For formula-fed infants, the formula should be enriched with iron [14];
  - On the one hand, iron supplementation in infancy reduces the risk of developing ID/IDA and its sequelae, but on the other hand, excess iron and its toxicity can delay growth and nervous system development, disturb trace element metabolism, increase susceptibility to infections, and disrupt development of the gut microbiota [209];
  - Introduce meat products from the age of 6 months; heme iron in meat is more available than non-heme iron, and increases the absorption of non-heme iron [210,211];
  - In all infants (<12 months of age), avoid administration of unmodified cow’s or goat’s milk [148];
  - In children aged 1–5 years, limit the supply of cow’s milk to 600 mL/day [140] and introduce at least three portions a day of iron-containing foods (e.g., meat, fortified breakfast cereals);
  - Include meat, fish, poultry, and foods that facilitate iron absorption (rich in vitamin C: citrus, tomatoes, potatoes) in the diet, and limit/eliminate foods that inhibit iron absorption such as tea, phosphates, and phytates, common in vegetarian diets [175].
8. Beyond the beneficial effect of iron supplementation, possible negative effects of such an intervention should also be borne in mind, in particular, the following [182]:
  - Excess oral iron has a negative impact on the gut microbiota, promoting the growth of pathogenic bacteria [212];
  - Excess iron, especially in iron-rich foods such as red meat and iron-fortified foods, may be a factor in the development of colorectal cancer [213]. Among other things, this is linked to the ability of iron to produce free radicals and promote the growth of cancer cells. Iron intake has been shown to correlate with the development of colorectal cancer [214,215]. For this reason, caution should be exercised and excessive and unnecessary iron supplementation should be avoided [216,217,218].
9. ID and IDA are relatively common in adolescent girls with heavy and/or prolonged menstrual bleeding. Despite this, recommendations for the management of this condition are inconsistent in terms of the indications for monitoring and treatment of ID and IDA in this group. As a result, ID/IDA remains undiagnosed and untreated in many girls [46]. According to a 2016 Cochrane systematic review (Low et al.) [219], daily iron supplementation effectively reduces the incidence of anemia and iron deficiency in menstruating women, increases hemoglobin levels and iron stores, improves exercise capacity, and reduces symptoms of fatigue. Gastrointestinal adverse effects are a negative consequence of such management. Furthermore, the results of another Cochrane review from 2020 (Fernández-Gaxiola et al.) [220] showed that intermittent iron supplementation in menstruating women can be an effective intervention to reduce anemia and improve hemoglobin levels compared to no treatment, placebo, or daily supplementation. Intermittent supplementation is also associated with fewer adverse effects compared to daily supplementation. The results were not affected by whether the preparations were given once or twice a week, for less or more than three months, and whether the iron dose was smaller or higher than 60 mg per week. The efficacy of this treatment was also not affected by the baseline hemoglobin concentration. Unfortunately, the conclusions drawn from this analysis are based on relatively low-quality research. This problem is also the main conclusion of the 2022 systematic review by Skolmowska et al. [221].

## 8. Summary

ID/IDA is a common health problem among children and adolescents and the most common hematological disorder that requires visits to the doctor. Despite this, there are still no well-conducted clinical trials in the pediatric population or reliable scientific evidence to unequivocally justify the treatment regimens used. For example, a meta-analysis by MacCann et al. [22] on the role of iron in brain development identified a large number of studies of low quality and considerable heterogeneity, with few studies directly addressing pregnancy and early infancy. In view of the inconsistent research findings on this issue, and despite the theoretical rationale, it is not possible to clearly determine the impact of ID on the development of the central nervous system (CNS) and on the child’s subsequent functioning. For this reason, further well-designed population-based studies are needed to answer questions about the prevention and treatment of iron deficiency based on scientific evidence, and any recommendations made should be treated with great caution. The lack of epidemiological data relating directly to the Polish pediatric population constitutes an additional difficulty.

The recommendations proposed in this document are based on available data from clinical and observational studies, but they also take into account many years of experience and practice in the management of ID/IDA in children. Furthermore, in clinical decision-making, one should be mindful of the risks that arise from chronic iron deficiency, especially in the first months and years of life, but at the same time, the potential toxicity of excess iron in the body and adverse effects of chronic iron supplementation should be taken into account.

## 9. Recommendations

The strength of evidence has been graded as follows:

1—strong recommendation (applies to the general population and all patients in most circumstances; benefits clearly outweigh risks).

2—weak recommendation (working group opinion agreed or for consideration; the best action may depend on the circumstances, benefits, and precisely balanced or uncertain risks).

The quality of evidence has been graded as follows:

(A) High: it is very unlikely that further research will change the current estimate of effect.
(B) Moderate: further research is likely to have an important impact on our confidence in the estimate of effect and may change the estimate
(C) Low: further research is very likely to have an important impact on our confidence in the estimate of effect and is likely to change the estimate.
(D) Very low: any estimates as to effects are very uncertain.

### 9.1. Recommendation No. 1: Diagnostic Criteria

- Iron deficiency (ID) in children: determine ferritin (SF) [1B] and possibly CRP (or another inflammatory marker) levels [2C]. The diagnosis of ID is mandated by SF levels below the lower limit for a given age (table below) unless there is concurrent inflammation. With inflammation, this standard should be increased to 30 µg/L in children under 5 years of age and to 70 µg/L in children over 5 years of age [2C]. Optimally, SF levels should be determined in a healthy state, without concomitant infections and other inflammation-mediated diseases.
- Iron deficiency anemia (IDA): criteria 1 and 2 met simultaneously:
  (1) hemoglobin (Hb) concentration below the age-specific normal range (Table 4) [1B] (severe IDA—Hb < 7 g/dL and/or poor general condition, e.g., hemodynamic disturbances, impaired consciousness, severe symptoms of anemia),
  (2) ID criterion met or reduced mean corpuscular volume (MCV) (provided thalassemia has been excluded) below the age-specific normal range (normal red blood cell indices do not exclude iron deficiency as the cause of anemia, so if there are clinical signs of ID or a strong suspicion of ID, further tests need to be performed) [2B].

### 9.2. Recommendation No. 2: Monitoring for ID/IDA

1. Assessment of signs and symptoms and risk factors for ID/IDA during screening tests, e.g., during periodic check-ups and immunizations [1A].
2. Laboratory diagnosis (complete blood count, ferritin, and possibly CRP levels) in all children who develop signs or symptoms of ID/IDA (Table 3) [1B].
3. Prophylactic laboratory tests in children at risk (Table 2) [2C]:
  - Preterm newborns: 1–2 months of age;
  - 9–15 months of age;
  - 2–5 years of age;
  - In girls at age 13 (or 1 year after menarche), and every 1–2 years thereafter;
  - In boys once in the period of intensive growth, during the pubertal spurt.

### 9.3. Recommendation No. 3: Therapeutic Management Following ID/IDA Diagnosis

**Figure 1:**
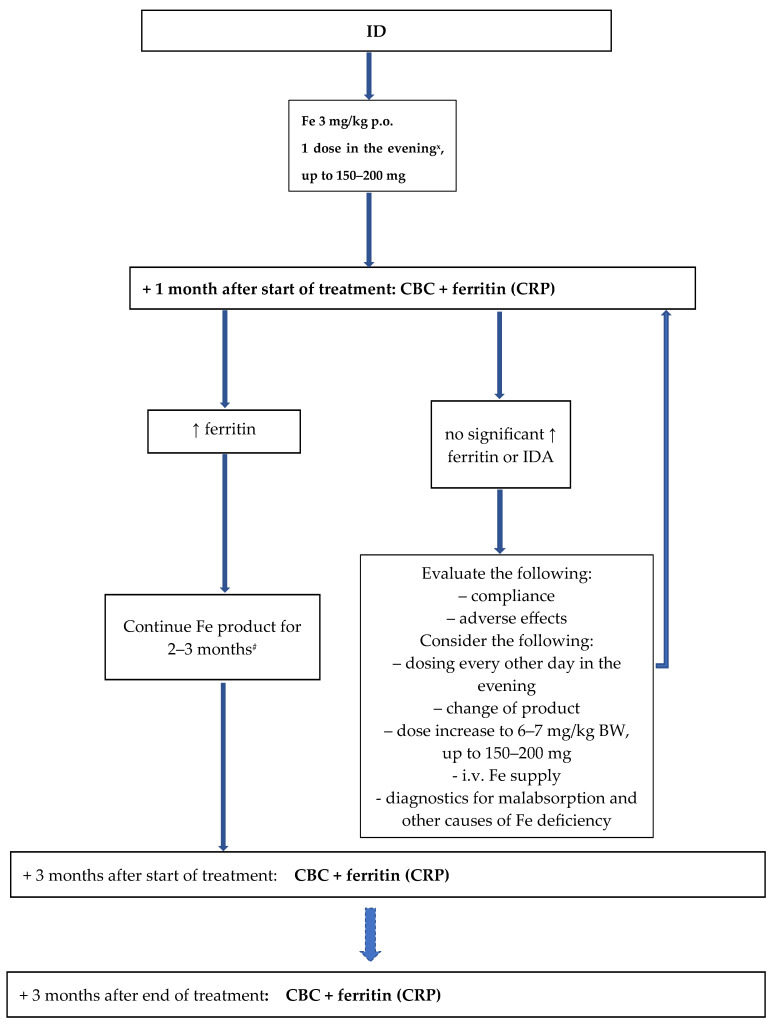
Management of iron deficiency in children. ^#^ If it is an oral preparation. ^x^ In selected cases, it is also possible to administer the daily dose in 2–3 divided doses. ↑—increase(d). CBC—Complete Blood Count. CRP—C-reactive protein. ID—Iron deficiency. IDA—Iron deficiency anemia. P.o.—per os. i.v.—Intravenous. BW—Body weight.

**Figure 2:**
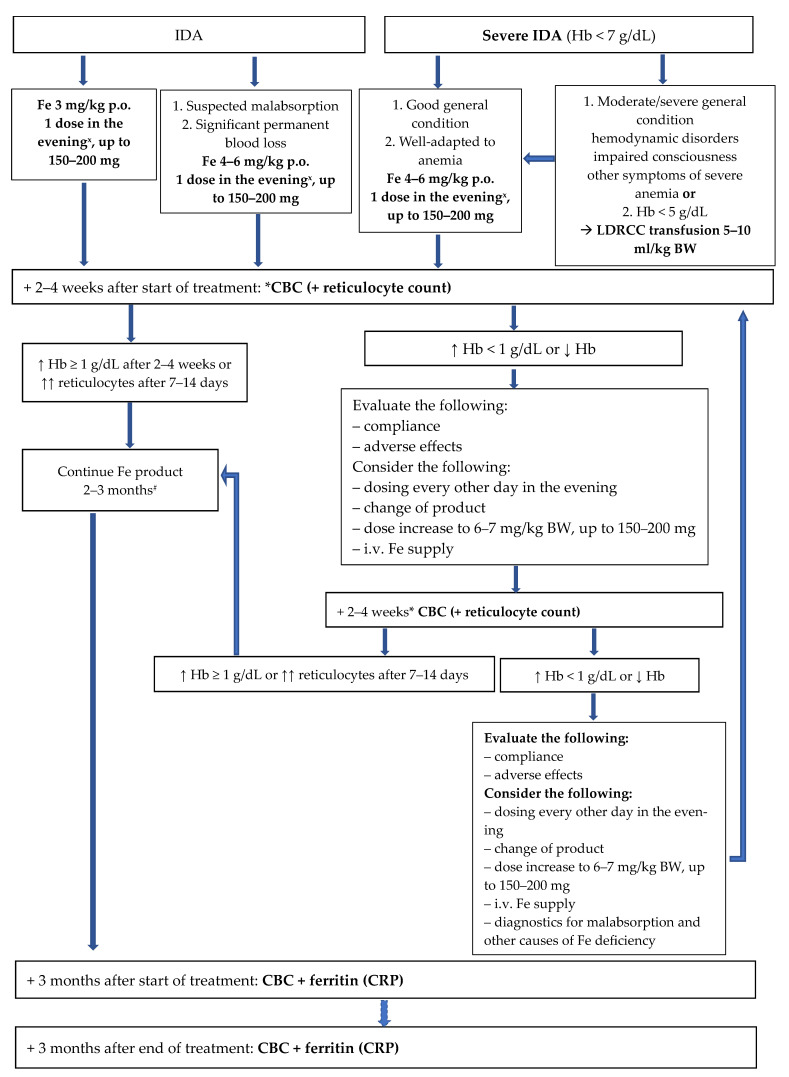
Management of iron deficiency anemia in children. * Or earlier if the child’s condition worsens, in which case perform CBC with reticulocyte count immediately. ^#^ If it is an oral preparation. ^x^ In selected cases, it is also possible to administer the daily dose in 2–3 divided doses. LDRCC—leukodepleted red cell concentrate. ↓—decrease(d). ↑—increase(d). CBC—Complete Blood Count. CRP—C-reactive protein. Hb—Hemoglobin. ID—Iron deficiency. IDA—Iron deficiency anemia. P.o.—per os. i.v.—Intravenous. BW—Body weight.

1. Diet modification, if necessary (Section 6.1) [1C].
2. For treatment regimens for ID and IDA in children, see Figure 1 and Figure 2 [1B].

### 9.4. Recommendation No. 4.: Prevention of ID/IDA

1. Delayed (>120–180 s) umbilical cord clamping should be used in all newborns [1A].
2. No indication for widespread iron supplementation in healthy breastfed infants and young children with normal birth weights. However, prophylactic iron supplementation may be recommended in neonates and infants born prematurely and at high risk of ID/IDA, if a low intake of iron-rich complementary foods is observed [1B].
3. Infants who are artificially fed up to 6 months of age should be given appropriate iron-fortified infant formulas with an iron content of 4–8 mg/L [2C].
4. Newborns and infants with low birth weights (2000–2500 g) should receive iron preparations at a dose of 2 mg/kg BW/day, starting at 2–6 weeks of age, until 6 months of age, regardless of whether they are pre-term or full-term newborns/infants [1B].
5. Newborns and infants with a birth weight of <2000 g should receive iron preparations at a dose of 2–3 mg/kg BW, according to the ESPGHAN guidelines for enteral nutrition in preterm infants [1B].
6. Follow-on formulas should be enriched with iron; there is insufficient evidence to determine the optimal iron concentration in follow-on formulas [2B].
7. From 6 months of age, all infants and young children should be given iron-rich solid foods, including meat products and/or iron-fortified foods [1C].
8. Unmodified cow’s milk should not be given before the age of 12 months, and in older children, its intake should be limited to <500 mL per day [1A].
9. Intermittent iron administration (at a total weekly dose of 50–100 mg) is recommended for girls with heavy/prolonged menstrual bleeding and a history of at least two episodes of ID/IDA requiring iron treatment. Similar treatment applies to children and adolescents permanently at risk of developing ID/IDA due to adherence to a low heme iron diet, practicing competitive sports, obesity, etc., with a history of at least two episodes of ID/IDA requiring iron treatment. In view of the low quality of evidence, the optimal dosage and duration of prophylactic supplementation cannot be determined [2B].

## Figures and Tables

**Table 1:** The most common causes of iron deficiency in children.

I. Insufficient Supply and/or Absorption of Iron from the Gastrointestinal Tract			
Improper Diet	Gastrointestinal Pathology	Increased Synthesis of Hepcidin	Increased Gastric pH
Plant foods and diets low in bioavailable iron Food selectivity and phobias in young children Weight loss (including anorexia) Feeding infants unmodified cow’s milk Diets high in iron absorption inhibitors, e.g., tannins (coffee, tea), fiber, phytic acid (oatmeal, cereal bran), oxalic acid (spinach, beans, nuts), polyphenols (vegetables, fruit, some cereals and legumes, tea, coffee, and wine), and calcium (dietary supplements, addition to fruit juices)	Celiac disease Inflammatory bowel disease: Crohn’s disease, ulcerative colitis Food allergies/food intolerance Enteropathy—leaky gut syndrome Short bowel syndrome Chronic diarrhea Anatomic malformations of the intestines (intestinal malrotation, volvulus) Giardiasis	Chronic and recurrent infections (e.g., urinary tract infections, respiratory infections) Chronic inflammation—allergic, autoimmune diseases Obesity IRIDA (iron refractory iron deficiency anemia)—mutation in TMPRSS6 gene—inadequate hepcidin secretion, resistance to iron treatment Practicing competitive sports	Excessive intake of antacid foods (which increase gastric pH), mainly milk (>600 mL/day) and dairy products Treatment with proton pump inhibitors and antacids Helicobacter pylori infection
**II. Chronic Blood Loss**			
Prolonged and/or heavy menstrual bleeding Food allergy Mucositis, gastric, and duodenal ulcers Inflammatory bowel disease Meckel’s diverticulum (especially in children with intussusception and with a clinical picture suggestive of appendicitis) Recurrent nosebleeds			

**Table 2:** Risk factors for iron deficiency and iron deficiency anemia.

Age-Related	Nutrition-Related	Others
Newborns and infants born prematurely	Plant foods and diets low in bioavailable iron	Gastrointestinal diseases and malabsorption disorders listed in Table 1
2. Newborns and infants treated with erythropoietin	2. Food selectivity and phobias in young children	2. Active inflammatory autoimmune diseases
3. Children from multiple pregnancies	3. Infants who were introduced to solid meat-containing foods too late	3. Chronic infections
4. Children of mothers with iron deficiency or iron deficiency anemia, diabetes mellitus, chronic disease, or pre-eclampsia	4. Infants fed unmodified cow’s milk before 12 months of age	4. Obesity
5. Newborns who are small for their gestational age (2000–2500 g)	5. Excessive milk supply in the diet (>600 mL/day)	5. Practicing competitive sports/intensive physical exercise
6. Infants with perinatal blood loss, fetal–maternal leakage, or twin-to-twin transfusion syndrome	6. Intensive weight loss (including anorexia)	6. Malnutrition
7. Adolescent girls with heavy/prolonged menstrual bleeding		

**Table 3:** Symptoms of iron deficiency and iron deficiency anemia.

Symptoms of Iron Deficiency			
General Symptoms	Neurodevelopmental Disorders	Neurological Disorders	Symptoms Related to Skin and Mucous Membranes
chronic weakness and fatigue	delayed psychomotor development [60,61,62,63,64]	attention-deficit/hyperactivity disorder (ADHD) [65,66]	angular cheilitis, glossitis, taste disorders, Plummer–Vinson syndrome (sideropenia with mucosal atrophy of the tongue, pharynx, esophagus), swallowing disorders [67]
poorer exercise tolerance	slower processing of visual and auditory stimuli [68,69,70,71,72,73,74]	restless leg syndrome (RLS) andperiodic limb movement disorder (PLMD) [66,75]	dry and rough skin
weakened immunity [76,77]	learning and memory difficulties [62,78,79]	affective apnea attacks in children aged 6–48 months [66,80]	koilonychia (spoon nails), increased brittleness of nails
reduced muscle function	cognitive impairment [19,81]	febrile convulsions (higher risk in children with low ferritin levels) [82]	hair loss, dry and damaged hair
impaired concentration		sleep disorders [83]	
irritability, psychomotor agitation, sleep disturbance			
decreased appetite			
pica (distorted appetite) [84,85]			
reduced physical capacity [86]			
**Symptoms of Iron Deficiency Anemia**			
General symptoms	Symptoms related to skin and mucous membranes	Cardiac symptoms	Other symptoms
hypersomnia	pallor of the skin and the oral and conjunctival mucosa	accelerated heart rate (tachycardia)	prolonged wound and tissue healing [87]
reduced exercise tolerance	stomatitis and glossitis [88]	functional heart murmur	thrombosis [89,90,91]
rapid fatigue, including reduced feeding time in newborns and infants		dyspnea	febrile convulsions [82]
headaches and dizziness		symptoms of circulatory insufficiency	
difficulty concentrating and remembering, poor academic performance [92]			
irritability			
slow development rate [93,94]			
loss of consciousness			

**Table 4:** Normal values of hemoglobin concentration, mean corpuscular volume (MCV), and ferritin concentration for the pediatric population (without associated inflammation).

Age	Hemoglobin [g/dL]	MCV [fL]	Ferritin [µg/L]
0–7 days	13.5–20	95–115	153–1092
8–30 days	10–16	85–100	247–692
1–3 months	9.5–14.5	85–100	148–744
4–9 months	9.5–13.5	75–95	21–240
9–24 months	10.5–13.5	75–85	10–168
2–16 years	11.5–15	77–85	10–99
>16 years, girls	12–16	78–95	18–103
>16 years, boys	13–17	78–95	16–213

**Table 5:** Foods with the highest iron content.

Meat-Based Foods with the Highest Iron Content (Bioavailability: 20–25%)	Plant-Based Foods with the Highest Iron Content (Bioavailability: 5–10%)	Products That Lower Stomach pH and Facilitate Iron Absorption
pork liver	wheat bran	orange juice
veal liver	sesame seeds	lemon juice
beef	soybeans	grapefruit juice
pork	lentils	apples
lamb	millet	grapes
game	white beans	gooseberries
egg yolk	dried apricots	pears
trout	spinach	raspberries
clams	chickpeas	
sardines	tofu	
mackerel	pumpkin seeds	
tuna	quinoa	
salmon	amaranth	
cod	sesame	

**Table 6:** List of iron products available in Poland, provided by a drug information website: www.lekinfo24.pl (accessed on 23 May 2023), based on SPCs and/or manufacturers’ information.

Brand Name	Form and Quantity of Iron	Additional Ingredients	Type	Patient Group
Oral Preparations				
**Elemental Iron**				
Feminovit Żelazo (Salvum Lab., Jelenia Góra, Poland)	film-coated tablets, 28 mg Fe (elemental iron)	vitamin C—40 mg	dietary supplement	adults
Innofer (Chiesi Poland, Warsaw, Poland)	oral suspension, 20 mg Fe/mL (elemental iron)	–	foods for special medical purposes	infants (including those with low birth weights), children, adolescents, and adults
Innofer Baby (Chiesi Poland, Warsaw, Poland)	oral suspension, 10 mg Fe/mL (elemental iron)	–	foods for special medical purposes	newborns (including preterm and those with low birth weights), infants, and children
Innofer (Chiesi Poland, Warsaw, Poland)	capsules, 20 mg Fe (elemental iron)	–	dietary supplement	children over 12 years of age and adults
**Iron II Salts**				
Ascofer (Espefa, Kraków, Poland)	film-coated tablets, 23.2 mg Fe II (iron II gluconate)	–	medicine (OTC)	children over 3 years of age, adolescents, and adults
Ascofer Plus (Espefa, Kraków, Poland)	film-coated tablets, 14 mg Fe (iron II gluconate)	folic acid (200 µg), vitamin C (80 mg)	dietary supplement	adults
Biorythm Żelazo (Stada, Warsaw Poland)	prolonged-release capsules, 28 mg Fe (iron II fumarate)	vitamin C (40 mg)	dietary supplement	adults
Chela-Ferr Bio-Complex (Olimp, Dębica, Poland)	capsules, 14 mg Fe (iron II diglycinate)	folic acid (200 µg), vitamin B_6_ (1.4 mg), vitamin B_12_ (2.5 µg), vitamin C (40 mg)	dietary supplement	children over 3 years of age, adolescents, and adults
Chela-Ferr Forte Olimp, Dębica, Poland)	capsules, 28 mg Fe (iron II diglycinate)	folic acid (400 µg), vitamin B_6_ (1.4 mg), vitamin B_12_ (2.5 µg), vitamin C (40 mg)	dietary supplement	adults
Chela Ferr Med Olimp, Dębica, Poland)	capsules, 30 mg Fe (iron II diglycinate)	–	foods for special medical purposes	children over 3 years of age, adolescents, and adults
Ferradrop (Aura Herbals, Sopot, Poland)	oral liquid, 14 mg Fe/15 mL (iron II diglycinate)	folic acid (100 µg/15 mL), vitamin B_6_ (0.7 mg/15 mL), vitamin B_12_ (1.25 µg/15 mL), vitamin C (40 mg/15 mL)	dietary supplement	adults
Sorbifer Durules (Egis, Budapest, Hungary)	prolonged-release tablets, 100 mg Fe II (iron II sulphate)	vitamin C (60 mg)	medicine (Rp)	adolescents above 12 years and adults
Szelazo+ SR (Lek-Am, Warsaw, Poland)	prolonged-release capsules, 28 mg Fe (iron II diglycinate)	folic acid (400 µg), vitamin B_6_ (1.4 mg), vitamin B_12_ (2.5 µg), vitamin C (40 mg)	dietary supplement	adults
Tardyferon (Pierre Fabre Médicament Polska, Warsaw, Poland)	prolonged-release tablets, 80 mg Fe II (iron II sulphate)	–	medicine (Rp)	children over 10 years of age and adults
Tardyferon-Fol (Pierre Fabre Médicament Polska, Warsaw, Poland)	modified-release film-coated tablets, 80 mg Fe II (iron II sulphate)	folic acid (350 µg)	medicine (Rp)	adults
Żelazo Apteo (Synoptis, Warsaw, Poland)	capsules, 30 mg Fe (iron II fumarate)	–	dietary supplement	adults
Żelazo Extra (Activlab, Bochnia, Poland)	effervescent tablets, 14 mg Fe (iron II diglycinate)	folic acid (200 µg), vitamin B_6_ (5 mg), vitamin B_12_ (10 µg), vitamin C (80 mg), zinc (10 mg), copper (1 mg)	dietary supplement	adolescents over 12 years of age and adults
**Iron III Salts**				
Actiferol Fe (Polski Lek, Wadowice, Poland)	powder, 7 mg Fe (iron III pyrophosphate)	–	dietary supplement	infants and children
Actiferol Fe (Polski Lek, Wadowice, Poland)	powder, 15 mg Fe (iron III pyrophosphate)	–	dietary supplement	infants, children, adolescents, and adults
Actiferol Fe (Polski Lek, Wadowice, Poland)	capsules, 30 mg Fe (iron III pyrophosphate)	–	dietary supplement	children, adolescents, and adults
Actiferol Fe (Polski Lek, Wadowice, Poland)	powder, 30 mg Fe (iron III pyrophosphate)	–	dietary supplement	children, adolescents, and adults
Actiferol Fe Forte (Polski Lek, Wadowice, Poland)	capsules, 30 mg Fe (iron III pyrophosphate)	folic acid (200 µg)	dietary supplement	adults
Actiferol Fe Krople (Polski Lek, Wadowice, Poland)	oral suspension, 2.5 mg Fe/5 drops (iron III pyrophosphate)	–	dietary supplement	infants, children, adolescents, and adults
Actiferol Fe Spray (Polski Lek, Wadowice, Poland)	oral spray, 3.5 mg Fe/application (iron III pyrophosphate)	–	dietary supplement	children over 3 years of age, adolescents, and adults
Actiferol Fe Start (Polski Lek, Wadowice, Poland)	powder, 7 mg Fe (iron III pyrophosphate)	folic acid (200 µg), vitamin B_6_ (1.4 mg), vitamin B_12_ (2.5 µg), vitamin C (20 mg)	dietary supplement	infants, children, adolescents, and adults
Fe-Lip Liposomal Iron 7 mg (Genexo, Warsaw, Poland)	oral gel, 7 mg Fe (ammonium iron III citrate)	–	dietary supplement	children over 1 year of age, adolescents, and adults
Fe-Lip Liposomal Iron 20 mg (Genexo, Warsaw, Poland)	oral gel, 20 mg Fe (ammonium iron III citrate)	–	dietary supplement	adults
Feroplex (Italfarmaco, Milan, Italy)	oral solution, 40 mg Fe III/15 mL (iron III protein succinylate)	–	medicine (Rp)	children over 1 year of age, adults
Ferovit Bio Special (Hasco-Lek, Wroclaw, Poland)	soft capsules, 28 mg Fe (iron III pyrophosphate)	folic acid (400 µg), vitamin B_6_ (1.4 mg), vitamin B_12_ (2.5 µg), vitamin C (40 mg)	dietary supplement	adults
Ferovit Bio Special Kids (Hasco-Lek, Wroclaw, Poland)	oral liquid, 7.5 mg Fe/2.5 mL (iron III pyrophosphate)	–	dietary supplement	children over 3 years of age
Ferrum Lek (Sandoz, Kundl, Austria)	syrup, 50 mg Fe III/5 mL (iron III hydroxide–polymaltose complex)	–	medicine (Rp)	infants, children, adolescents, and adults
Ferrum Lek (Sandoz, Kundl, Austria)	chewable tablets, 100 mg Fe III (iron III hydroxide–polymaltose complex)	–	medicine (Rp)	adolescents above 12 years and adults
Sideral (Pharmanutra, Piza, Italy)	capsules, 14 mg Fe (iron III pyrophosphate)	vitamin B_12_ (0.375 µg), vitamin C (60 mg)	dietary supplement	adults
Sideral Folic (Pharmanutra, Piza, Italy)	powder, 21 mg Fe (iron III pyrophosphate)	folic acid (400 µg), vitamin B_6_ (1 mg), vitamin B_12_ (1.75 µg), vitamin D (10 µg), vitamin C (70 mg)	dietary supplement	children over 3 years of age and adults
Sideral Forte (Pharmanutra, Piza, Italy)	capsules, 30 mg Fe (iron III pyrophosphate)	vitamin C (70 mg)	dietary supplement	adults
Sideral Gocce (Pharmanutra, Piza, Italy)	oral drops, 7 mg Fe/mL (iron III pyrophosphate)	–	dietary supplement	children over 3 years of age and adults
**Iron Salt Combinations**				
Biofer (Pharbio/Orkla, Warsaw, Poland)	tablets, 13.5 mg Fe (heme iron + iron II diglycinate)	vitamin C (40 mg)	dietary supplement	adolescents over 12 years of age and adults
Biofer Folic (Pharbio/Orkla, Warsaw, Poland)	tablets, 13.5 mg Fe (heme iron + iron II diglycinate)	folic acid (200 µg), vitamin C (40 mg)	dietary supplement	adolescents over 12 years of age and adults
Ibuvit Żelazo + C (Polpharma, Starogard Gdański, Poland)	gradual-release tablets, 20 mg Fe (rapid-release layer, iron II diglycinate; normal-release layer, iron II sulphate; delayed-release layer, iron III pyrophosphate)	vitamin C (70 mg)	dietary supplement	adolescents above 12 years and adults
**Parenteral Preparations**				
CosmoFer (Pharmacosmos, Holbaek, Denmark)	injections, 50 mg Fe III/mL (iron III hydroxide–dextran complex)	–	medicine (Lz)	adults and adolescents over 14 years of age
Diafer Pharmacosmos, Holbaek, Denmark)	injections, 50 mg Fe III/mL (ferric derisomaltose)	–	medicine (Rp)	adults
Ferinject (Vifor, Neuilly-sur-Seine, France)	injections, 50 mg Fe III/mL (ferric carboxymaltose)	–	medicine (Rp)	adults and children over 1 year of age
Ferrum-Lek (Sandoz, Kundl, Austria)	injections, 50 mg Fe III/mL (iron III hydroxide–dextran complex)	–	medicine (Rp)	adults and children over 4 months of age
Monover (Pharmacosmos, Holbaek, Denmark)	injections, 100 mg Fe III/mL (ferric derisomaltose)	–	medicine (Rp)	adults
Venofer (Vifor, Neuilly-sur-Seine, France)	injections, 20 mg Fe III/mL (iron III hydroxide–sucrose complex)	–	medicine (Lz)	adults and children

Product type labeling: medicine (Rp)—a medicinal product included in the availability category of prescription drugs; medicine (Lz)—a medicinal product included in the availability category of inpatient-only medicines; medicine (OTC)—a medicinal product included in the availability category of non-prescription (over-the-counter) medicines; dietary supplement—a foodstuff supplementing the normal diet, a concentrated source of vitamins, minerals, or other substances, single or combined, in dosage form; foods for special medical purposes—a category of food intended for patients requiring special dietary management under medical supervision.
